# Self-Esteem and National Identification in Times of Islamophobia: A Study Among Islamic School Children in The Netherlands

**DOI:** 10.1007/s10964-018-0906-6

**Published:** 2018-08-10

**Authors:** Jochem Thijs, Lisette Hornstra, Fatima Zohra Charki

**Affiliations:** 1 Ercomer, Department of Interdisciplinary Social Science, Utrecht University, Padualaan 14, 3584 CH Utrecht, The Netherlands; 2 Department of Education, Utrecht University, Heidelberglaan1, 3508 TC Utrecht, The Netherlands

**Keywords:** Islamic schools, Muslim children, Discrimination, Teachers, Self-esteem, National identification

## Abstract

Despite strong debates about the role of Islamic education in Western societies, very little is known about the ways these schools can affect how Muslim children feel about these societies and themselves. This research examined how the self-esteem and national identification of Islamic schools students in a non-Muslim country (*N* = 707; *M*_*age*_ = 10.02; *SD* = 1.25; 56.9% girls) depend on their perceptions of religious discrimination and the student-teacher relationship, as well as their teachers’ religious background and implicit religious attitude. Children reported substantially more religious discrimination against their group than against themselves. Religious discrimination was associated with lower self-esteem and weaker national identification, whereas a close bond with the teacher was associated with higher self-esteem and stronger national identification. Children with a non-Muslim teacher reported more national identification than students with a Muslim teacher, but less so if this teacher had a comparatively positive attitude toward Muslims. Results provide insights on how self-esteem and national identification can be encouraged within the context of Islamic education.

## Introduction

Muslims are among the most discriminated groups in current day Western-Europe where many people hold the view that Islam is an alien religion and incompatible with mainstream values (Hagendoorn and Sniderman 2007; Strabac and Listhaug 2008). Due to this Islamophobic climate, it can be rather difficult for Muslim children to grow up there. An increasing number of these children attend Islamic schools (Dronkers 2016; Maussen and Bader 2014) that aim to provide them with a safe environment to develop and express their religious identity, and a shelter against religious discrimination. Islamic schools allow children to feel good about their religious background and thus about themselves. However, despite this “safe haven” function, the role and impact of Islamic education in Western countries have not gone undisputed. Critics have argued that Islamic schools may undermine social cohesion and promote segregation by making children ill prepared to function in non-Muslim or secular societies, or worse, by turning them away from these societies altogether (Elbih 2012; Hussain and Read 2015; Zine 2007). Yet to date, there are very few empirical findings to support or reject these fears and criticisms.

The present research makes a unique contribution to the literature by examining how teachers in Islamic schools can affect both the self-esteem and the national identification of their Muslim students (aged 8–14 years). Both self-esteem and national identification are crucial adjustment outcomes for children who live in a country where their religious group is highly stigmatized. Rather than comparing the impact of Islamic versus non-Islamic education, the study investigates how children’s self-esteem and national identification are associated with teacher-related factors *within* Islamic schools, and it examines whether these factors can counteract the (anticipated) negative impact of children’s perceptions of religious discrimination directed against themselves (personal discrimination) and their peers (group discrimination). As such, this research could inform practical attempts to help Islamic school students cope with discrimination and to promote high self-esteem and a sense of identification with a country where many people are negatively inclined toward their religion.

The study used a quantitative approach and sampled 35 primary school classes from around a third of all Islamic schools in the Netherlands. Although Christianity was and is the dominant religion in the Netherlands, it is one of the most secular countries in the world (De Graaf and Te Grotenhuis 2008). However, the country also has a substantial Muslim population, and since the 1980’s, Islamic schools were established there in order to provide Islamic instruction and to raise the academic performance of Muslim students (Merry and Driessen 2016). In the Dutch school system, primary school children tend to have one or two teachers the whole year round, and this makes these teachers significant adults in their daily lives. As teachers can function as secondary attachment figures for primary school children (Verschueren and Koomen 2012), children’s perceptions of the student-teacher relationship were examined. Many teachers in Muslim schools are non-Muslim teachers with a Dutch background (Driessen and Merry 2006; Dronkers 2016). These non-Muslim Dutch teachers could potentially act as “a bridge to Dutch society” for children in Islamic schools who may otherwise only have limited contact with native Dutch or non-Muslim people (Driessen and Merry 2006). As such, it was examined whether a close relationship with the teacher, especially a non-Muslim one, is associated with Muslim students’ self-esteem and national identification. Additionally, the role of the teachers’ implicit attitude toward Muslims versus non-Muslims was examined. As will be explained, this variation in religious background and/or attitude may have important implications for the national identification and self-esteem of Muslim children in a non-Islamic country such as the Netherlands.

### Personal and Group Discrimination, Self-Esteem, and National Identification

Research has shown that children report less ethnic discrimination when they have fewer ethnic out-group classmates (Thijs, Verkuyten and Grundel 2013). In Islamic schools, all children are Muslim and this means that they are protected from religious peer discrimination in their school environment. Still, discrimination can take place outside schools, and Muslim children can be aware of prejudice directed toward their group even if they are not discriminated themselves. Research has shown that minority youths’ perceptions of personal and group discrimination are considerably related but also that they perceive more discrimination against their fellow group members than against themselves (Armenta and Hunt 2009; Verkuyten and Thijs 2002), a phenomenon known as the Personal/Group-Discrimination Discrepancy (PGDP; Taylor et al. 1990). This discrepancy was expected to be present in the current study as well.

Discrimination signals that an important part of the self, i.e., the group one belongs to, is negatively regarded and not accepted by others (Schmitt and Branscombe 2002), and this is especially problematic for young people who have to find their place in society (Schmitt et al. 2014). In forming their opinions about the self, children are strongly dependent on others, and researchers have documented a process of “reflected appraisals” whereby their evaluations of themselves are partly based on (their perceptions of) others’ opinions about them or the groups they belong to (Harter 1999). Numerous studies have shown that perceived discrimination has detrimental effects on the self-esteem of disadvantaged minority youth but the effect of personal discrimination is typically more pronounced than that of group discrimination (for a meta-analysis, see Schmitt et al. 2014). Both personal and group discrimination convey negative messages about one’s group membership which children may to come to internalize. Still, personal discrimination may have more negative implications for the self than group discrimination. Although group discrimination implies a devaluation of the group one belongs to, it is not directly targeted at the self. In fact, Muslim children may feel better about themselves if they perceive more discrimination against their religious in-group peers versus themselves, because it indicates that others’ opinions of them as individuals are relatively positive (“I must be really nice if they are so often discriminated while I am not”; see Bourguignon et al. 2006). This positive effect may counteract the negative impact of the devaluation of their religious group.

In countries where the majority is non-Muslim and Islam is regarded with suspicion by substantial parts of the population, perceived religious discrimination could also threaten the national belonging of Muslim school students. According to the so-called rejection-disidentification model (Jasinskaja-Lahti and Liebkind 2009), people tend to psychologically withdraw (disidentify) from groups they feel rejected by, and research has supported this model by showing that perceived discrimination undermines the national identification of Muslim adults (Maliepaard and Verkuyten 2018) and youth (Fleischmann and Phalet 2018) in Western societies. This previous research has not compared the effects of personal and group discrimination. However, it can be anticipated that Muslim children’s sense of national identification is more affected by the latter by the former than the latter. However painful individual experiences with discrimination can be, these experiences may be temporary and the future might look less pale. However, the impression that fellow group members are discriminated against indicates that anti-Muslim prejudice is widespread in society, and thus can be expected to have stronger implications for children’s national belonging (cf., Stevens and Thijs 2018).

### The Importance of the Student-Teacher Relationship

There is ample evidence that the quality of the student-teacher relationship is crucial for the academic, psychological, and social adjustment of primary school children (e.g., Roorda et al. 2011; Rudasill et al. 2010). Much of this positive impact can be explained from a so-called extended attachment perspective, which claims that teachers can function as secondary or surrogate attachment figures to children who can provide them with the necessary security and confidence to approach their worlds and with emotional support in times of stress (Verschueren and Koomen 2012). Children who share warm and close bonds with their teachers learn that they are socially accepted and worthy of love and affection, and research has shown that these relations promote the development of high self-esteem (Ryan et al. 1994; Verschueren et al. 2012). Additionally, there is some evidence that positive relationships with teachers can protect against the negative effects of discrimination. Trust in their teachers makes children more resilient in dealing with stressful life events (Pianta et al. 2003), and a longitudinal study among immigrant adolescents in Sweden found that personal ethnic peer harassment predicted lower self-esteem over time, but also these effects were not significant for students who reported positive bonds with their teachers (Özdemir and Stattin 2014). Thus, it was expected that Muslim children who experience more closeness in the relationship with their teachers have higher self-esteem, and also that their self-esteem is less negatively affected by their perceptions of discrimination.

Additionally, the current study tested whether the student-teacher relationship plays these promotive and protective roles (see Motti-Stefanidi and Masten 2013) for children’s national identification. Although Islamic schools are not representative of the dominant culture in Western societies, they are state funded and part of the national educational system (Dronkers 2016) and thus expected to prepare their students to participate in the larger society (Barrett 2007). Accordingly, it is reasonable to anticipate that Islamic school students who share a positive, high-quality relationships with their teachers experience a stronger connection to their schools and thus to the country they live in. It can also be expected that the national identification of these students is less strongly affected by their perceptions of discrimination. The reason is that these positive bonds with their teachers will provide children with the confidence and security to cope with the prejudice against their group, and thus undermine their tendency to withdraw from the national group. These hypotheses have never been systematically investigated but they are clearly consistent with findings by Hussain and Read (2015). These authors conducted an extensive field study at three Islamic schools in the US and the UK, and they concluded that these schools can facilitate rather than undermine the future integration of their students by “giving them the confidence to interact with those from outside of their faith community” (p. 563).

### Teachers’ Religious Background and Implicit Religious Attitude

In addition to the degree of closeness that children experienced with their teachers, this study examined the religious background and the implicit religious attitude of the latter. Teachers in Islamic schools are not all Muslims, and they can differ in their attitude toward Muslims and Islam. Indeed, research among a large sample of primary and secondary school (public and private) teachers in Flanders (Belgium) found considerable between-teacher variation in the attitude toward Muslim students. This variation was only partly explained by teachers’ own religious background (Agirdag et al. 2012), which implies that both factors are sufficiently independent to include as separate predictors.

Although there is no reason to expect that the religious background of the teachers would relate to the self-esteem of Islamic school students, it probably affects their sense of national identification. Muslim children who grow up in a non-Islamic country have to develop a national identity that is shared with others who either are not religious or have different religious beliefs. According to the Common In-Group Identity Model (Dovidio et al. 2007), the development of such a shared identity would be stimulated by contacts with these other people. Students in Islamic schools have no opportunities to interact with non-Muslim classmates, but they can have a non-Muslim teacher. Research in Dutch non-Islamic primary schools has found that ethnic minority children who shared positive relationships with their ethnic majority teachers were more likely to think positive about the ethnic majority group in general, especially when they had few majority classmates to interact with (Thijs and Verkuyten 2012). This finding is consistent with Intergroup Contact Theory (Allport 1954; Pettigrew 1998) and indicated that, in the absence of out-group peers, teachers can be important contact figures for their students. Hence, in the present study, it was tested whether Islamic school students with a non-Muslim teacher would have stronger national identification compared to their peers with a Muslim teacher.

Next, given the strong debate surrounding the position of Islam, it might be difficult for teachers to express what they really think about Muslims, especially if they work at an Islamic school. The present study therefore used an implicit association test (IAT; Greenwald et al. 1998) to assess teachers’ attitude toward Muslims versus non-Muslim natives. The advantage of implicit attitude measures compared to self-report measures, such as questionnaires, is that they are less sensitive to socially desirability concerns. In a variety of domains, implicit measures are found to add to the prediction of variations in human behavior that are not accounted for by self-report measures (for an overview, see Greenwald et al. 2009). Especially with regard to controversial topics, such as ethnic or religious prejudice, implicit measures appear to be more predictive of subsequent behavior (Greenwald et al. 2009). In the domain of education, earlier studies have shown that the implicit attitudes of teachers can have important consequences for their students (Peterson et al. 2016; van den Bergh et al. 2010; Vezzali et al. 2012). For instance, teachers’ implicit ethnic prejudice has been found to affect ethnic minority students’ achievement (Van den Bergh et al. 2010) as well as students’ own attitudes toward other ethnic groups (Vezzali et al. 2012).

The very reasoning that predicts negative effects of perceived discrimination on children’s self-esteem also predicts a positive impact of teachers’ implicit attitude. Islamic school teachers who are favorably inclined toward Muslims are more likely to demonstrate this favorability in the behaviors and communications with their students. And such positive messages about their group can promote the self-esteem of Muslim children via the process of reflected appraisals (Harter 1999). Yet, and probably inadvertently so, the implicit religious attitude of Islamic school teachers may also undermine the national identification of their students. The reason is that a positive evaluation of Muslims versus non Muslims also implies a comparatively less positive evaluation of the latter, and in Western countries the majority of the population belongs to that group. As teachers’ beliefs and practices can affect their students’ beliefs toward society (e.g., Banks 2001), teachers with a comparatively less positive attitude toward non-Muslim natives may communicate this attitude to their students. Thus it is possible that teachers’ implicit attitude toward Muslims versus non-Muslims diminishes their students’ sense of national identification.

It is also important to consider the interaction between teachers’ religious background and implicit religious attitude. According to Self-Categorization Theory (Turner, Hogg, Oakes, Reicher and Wetherell, 1987) people are more likely to rely on in-group members than on out-group members to provide them with relevant information about the social world. And this would imply that the anticipated effects of teachers’ implicit attitude on children’s self-esteem and national identification would be stronger for Muslim versus non-Muslim teachers. Yet it also thinkable that children are more focused on non-Muslim teachers’ (implicit) communications about Muslim versus non-Muslims because such communications are informative about what the “outside world” thinks of their group. As a consequence, the implicit attitude of non-Muslim teachers could be more influential than that of Muslim teachers. Both possibilities were explored in this study.

## Current Study

The goal of the present research was to examine the antecedents of global self-esteem and national identification in Islamic school children (aged 8-14 years) in order to provide insights on how these aspects of psychological and social adjustment can be encouraged within this specific population. The study investigated children’s perceptions of religious discrimination against themselves and against other Muslim children. Both perceptions were anticipated to be related, and to prevent collinearity problems it was decided to combine them into a measure for *perceived overall discrimination* (the average of both perceptions) and a measure for the *group/personal discrepancy* (the difference between the group and personal discrimination perceived).^1^ The first expectation was that children would perceive more religious discrimination against their group than against themselves (H1). Next, it was expected that children’s overall perceptions of religious discrimination would be associated with lower self-esteem (H2a) and lower national identification (H3a), but also that the discrepancy between group and personal discrimination would be negatively related to self-esteem (implying a weaker effect of group versus personal discrimination; H2b) and positively related to national identification (implying a stronger effect of group discrimination; H3b). The study also investigated how teacher characteristics could compensate for and protect against these anticipated negative effects by testing whether children who shared a close relationship with their primary teacher would have higher self-esteem (H4) and stronger national identification (H5), and that closeness would diminish the negative effects of perceived overall discrimination on these variables (H6a and H7a, respectively), and thus also the anticipated effects of the discrepancy (H6b and H7b, respectively). Additionally, it was expected that children would report a stronger sense of national identification if their teacher was a non-Muslim (H8), as well as higher self-esteem (H9) and a weaker sense of national identification (H10) if their teachers’ attitude toward Muslims (versus non-Muslim natives) was comparatively positive. It was also explored whether the impact of teachers’ implicit attitude interacted with their religious background. Finally, children’s ethnic background, gender, and grade level were controlled for in all analyses.

## Method

### Participants

Participants were 707 students from 35 classes in 10 Islamic primary schools in the Netherlands. The mean age of the students was 10.02 years (*SD* = 1.25; range 8–14 years) and they were in the upper grades of primary school, i.e., grade 3 (31.7%), 4 (21.5%), 5 (27.0%), or 6 (19.5%). Age and grade level were strongly related (*r* = 0.85). Of these children, 56.9% were girls. The largest immigrant groups in the Netherlands and in Islamic schools are people with Turkish or Moroccan backgrounds (Central Bureau of Statistics 2016). Accordingly, in our sample, it was found that according to their ethnic self-definition, 43.0% of these children identified as Moroccan, 28.4% as Turkish, 1.4% as Dutch, and 23.5% as having another ethnicity. Ethnic self-definition was not reported by 3.6% of the students. For 10.9% of students, the reported ethnicity of their mother was different than the reported ethnicity of their father. Most of these children identified with their father’s ethnicity (87.8%). Most Turkish and Moroccan primary school children in the Netherlands are second or third generation immigrants (Dutch Inspectorate of Education 2018), and the large majority of respondents (77.8%) reported that Dutch was spoken at home. The classroom teachers (*N* = 35) of these students also participated. Most teachers were female (85.7%). Their mean age was 32.94 years (*SD* = 6.37). On average they had 7.51 years (*SD* = 5.77) of teaching experience with an average of 4.83 years (*SD* = 4.34) teaching experience at their current school. According to their ethnic self-definition, 42.9% of the teachers identified as Dutch, 20.0% as Turkish, 28.6% as Moroccan, and 8.7% as having another ethnic background. Twenty teachers reported to be Muslim, the other 15 teachers considered themselves to be non-Muslim.

### Procedure

At the time of the study (the spring of 2017) there were 49 Islamic primary schools in the Netherlands. As six of them were known to have Muslim teachers only, the other 43 were approached to participate. Eventually, 10 schools participated, as 14 schools couldn’t be properly contacted, 15 schools refused or didn’t respond after first contact, and four responded positively but too late. Data collection took place during the second semester of the school year (i.e., March and April). The schools were visited by one of the researchers. Beforehand, teachers were informed about the study and asked for their consent to participate (active consent). Parents also received a letter to inform them about the study and could object to participation of their child (passive informed consent). Beforehand, active informed consent was obtained from teachers and passive informed consent from parents. The parents of twelve children objected to participation. During data collection, students first received an introduction explaining the general purpose of the study, how to fill out the questionnaire, and it was explained that their anonymity was guaranteed. They completed the questionnaires during regular class hours. The questionnaires were part of a larger investigation and also included some additional scales in addition to the scales of interest of the present study. The questionnaire started with questions on demographic information and continued with other scales, including the measures on group discrimination, self-esteem, national identification, and the relationship with the teacher. While the students completed their questionnaires, their teachers were administered an implicit association test (IAT) and filled out questions on their background characteristics.

### Measures

#### Perceived discrimination

To assess children’s perceptions of *personal* and *group discrimination* we asked them the same three questions in relation to themselves and other children: “Are [respectively, you/ other children] ever called names because [you/they] are Muslim?”, “Do people ever act mean toward [you/other children] because [you/they] are Muslim?”, and “Are [you/other children] ever bullied because [you/they] are Muslim?”. These items were based on previous research on children’s perception of discrimination in the Netherlands (Verkuyten and Thijs 2002) and their response scales ranged from 1 (*No*) to 5 (*Yes!*). Principal components analyses indicated that the items loaded on two factors that explained 73.30% of the variance and corresponded to measures for personal discrimination (Cronbach’s alpha was 0.89) and group discrimination (Cronbach’s alpha was 0.82). However, both measures were strongly related (*r* = 0.60) and the Kaiser criterion indicated a single principal component explaining 58.26% of the variance. Thus the analyses (see below) included a score for children’s overall perceptions of discrimination (average of group and personal) as well as a discrepancy score (group minus personal).

#### Self-esteem

Children’s self-esteem was assessed with five items adapted from Harter’s Self-Perception Profile for Children, which is an established, reliable and valid self-concept measure (Harter 1999). Sample items are “Some children are happy with the way they are. How about you?” and “Some children are very satisfied with the way they do things. How about you?”. Agreement with the items was rated on a scale ranging from 1 (*No!*) to 5 (*Yes!*). Cronbach’s alpha was 0.74 for this scale, and the items loaded on one factor explaining 51.0% of the variance.

#### National identification

National identification was measured with six items that based on earlier research among preadolescent children in the Netherlands (Verkuyten 2002; Verkuyten et al. 2014). Three of these items focused on identification with the Netherlands as a country (“Do you feel at home in the Netherlands?”, “Are you proud of the Netherlands?”, and “Do you like it in the Netherlands?”) and the other three on their identification with the people living there (“Do you feel like a Dutch person?”, “Are you proud if a Dutch person wins a gold medal?”, and “Do you like to hear good things about Dutch people?”). Responses were rated on a five-point scale ranging from 1 (*No!*) to 5 (*Yes!*) and Cronbach’s alpha was 0.79. Although the term “Dutch” applies to all citizens of the Netherlands, it is sometimes used to denote its “original” inhabitants (ethnic Dutch). For children with a migration background (over 98 % of our sample) this could mean that their identification with the country may not run parallel to their identification with the people (see Barrett 2007). However, all items loaded on a single component that accounted for 50.2% of the variance. Thus, the participating children made no clear distinction between their identification with the Netherlands and Dutch people, and we examined national identification as unidimensional variable.

#### Closeness with teacher

To assess students’ perceptions of closeness in the relationship with their teachers they completed the *closeness* subscale of the Student Perception of Relationship with Teacher Scale (SPRTS; Koomen and Jellesma 2015). This scale consists of six items (including “I feel comfortable with my teacher” and “if I have a problem I can go to my teacher”). The response scale ranged from 1 (*No!*) to 5 (*Yes!*), Cronbach’s α was 0.84 and the items loaded on one factor explaining 56.7% of the variance. Previous research has provided support for the internal and external validity of the measure in a sample of Turkish-, Moroccan-, and native Dutch students in the upper grades of primary school (Thijs and Fleischmann 2015).

#### Implicit attitude toward Muslims versus non-Muslims

Teachers’ implicit attitude toward Muslims versus non-Muslims was assessed using an implicit association task (IAT) (Greenwald et al. 1998), which was based on the IAT by Van den Bergh et al. (2010). This response latency measure was administered on a laptop, using Inquisit software (by Millisecond). The IAT measured the relative strength of the association between religion (i.e., Muslim or non-Muslim) and the valence of words (i.e., the positive versus negative connotations of words). Respondents had to classify children’s names as “Muslim” (e.g., Hassan, Abdel) or “Non-Muslim” (e.g., Joost, Daan)^2^. The IAT consisted of seven blocks: three practice blocks and four test blocks. In the first block, the teachers were shown a series of single words that appeared in the middle of the screen (e.g., peace, war, paradise, fear) and had to classify each word as “good” by pressing the E key on the laptop or “bad” by pressing the I key on the laptop. In the second block, various names appeared on the screen and had to be classified as belonging to a Muslim by using the E key or belonging to a non-Muslim by using the I key. In the third and fourth blocks, which were the first test blocks, the aforementioned word and name categories were paired. When a word or name appeared on the screen, the presence of a positive meaning or a non-Muslim name had to be responded to by using the E key; the presence of a negative meaning or a Muslim name had to be responded to by using the I key. In the fifth block, only names had to be classified again but now by using the E key for a Muslim names and the I key for a non-Muslim name. In the sixth and seventh block, which were the again test blocks, the word and name categories were also paired but now in the opposite manner: Words with a positive meaning and Muslim names had to be responded to by using the E key, while words with a negative meaning and non-Muslim names had to be responded to by using the I key. The order of the blocks was counterbalanced across respondents. Response latencies were recorded for each response. Only the data for the test blocks were then used to calculate the IAT scores. The response latencies for the test blocks in which the participants had to respond similarly to “good” and “Muslim”, on the one hand, and “bad” and “Non-Muslim”, on the other hand, were then compared to the response latencies of test blocks in which the participants had to respond similarly to “bad” and “Muslim”, on the one hand, and “good” and “non-Muslim”, on the other hand. The assumption is that greater difficulties with the association of two particular categories (i.e., “bad” and “non-Muslim”) will produce longer response times for these pairs when compared to other pairs. In line with Greenwald et al. (1998), the error rate was about 4%. No participants had to be removed because of excessively high error rates. The raw data were transformed using the improved scoring algorithm of Greenwald et al. (2003). The standardized score (*d*) was then taken to be an indicator of a teacher’s implicit attitude toward Muslims versus non-Muslims. Higher scores indicated a greater preference for Muslims over non-Muslims.

### Data Analysis

To take the hierarchical structure of the data into account (students nested in classes), multilevel analyses were performed in MPlus in conjunction with the robust maximum likelihood (MLR) estimator (Version 7.4; Muthén and Muthén 2017). Multivariate models were specified to simultaneously include both dependent variables (self-esteem and national identification), and missing data were estimated using the Full Information Maximum Likelihood (FIML) method. The percentage of missing values varied from 0.0 to 13.6% for the different items included in the present study. Missing values were similarly distributed across students with varying characteristics (e.g., gender, grade) and missingness was not systematically related to scores on other variables included in the present study. The data were therefore considered to be missing at random, which can be accounted for by the FIML method (see Schafer and Graham 2002).

Prior to the analyses, the distribution of variance across the individual and class level was examined by calculating the intraclass correlations (ICCs) of the variables. Thereafter, a model (Model 1) was estimated which included the covariates students’ gender, ethnicity, and grade level^3^ as predictors of self-esteem and national identification (Model 1). In a subsequent model (Model), all level 1 predictors (overall discrimination, group minus personal discrimination, closeness, and interactions between the discrimination measures and closeness) were added according to the specified hypotheses. Next, in Model 3, all level 2 predictors (teachers’ religion, implicit religious attitude, and the interaction between both variables) were added. All categorical child predictors were entered as dummy variables, and a contrast was specified for teacher religion (“0.5” for Moslim versus “-0.5” for non-Muslim). Continuous variables were grand-mean centered before they were entered to the models. The significance of the coefficients for the different predictor variables was tested using Wald tests (z tests). The set level of significance was 5%. Model fit was evaluated with the Akaike Information Criterion (AIC) and by the Standardized Root Mean Square Residual (SRMR). The latter measure is calculated separately for the within and between level. Lower AIC values indicate better fit of subsequent models and SRMR values smaller than 0.1 are considered acceptable (Kline 2005).

## Results

### Preliminary Analyses

Table 1 contains the descriptive statistics and intercorrelations for the main variables. The correlations of teachers’ religion and implicit attitude toward Muslims versus non-Muslims with the other variables were estimated at the classroom level (*N* = 35). All other correlations were calculated at the individual student level.

**Table 1:** Intercorrelations, means, standard-deviations, and intraclass correlations for main study variables

	*N*	1.	2.	3.	4.	5.	6.	7.	8.	*M*	*(SD)*	ICC
1. Personal discrimination	635									1.81	(1.01)	.06*
2. Group discrimination	595	.60***								2.29	(1.09)	.04
3. Overall discrimination	654	.89***	.90***							2.03	(.93)	.04
4. Group/personal discrepancy	577	−.39***	.50***	.08						0.45	(.95)	.08*
5. Closeness with teacher	704	−.10*	−.08	−.11**	.04					3.84	(.91)	.08**
6. Self-esteem	652	−.26***	−.18**	−.25***	.05	.33***				4.54	(.62)	.06*
7. National identification	654	−.11**	−.14**	−.15***	−.03	.19***	.13**			3.28	(.93)	.03
8. Teacher religion (0.5 = Muslim; −0.5 = non-Muslim)	35	−.43*	.16	−.16	.51**	−.11	−.05	−.45**		0.07	(.50)	-
9. Teacher’s implicit religious attitude	35	−.31	.03	−.15	.33	−.10	−.08	−.49**	.52**	0.21	(.55)	-

*Note*: The number of respondents varies for the different measures of discrimination. The overall discrimination score was calculated if participants completed at least four of out the six discrimination items, whereas the other discrimination scores were calculated only if the respondents completed all of the items

*p < .05, **p < .01, ***p < .001

As anticipated, and consistent with the first hypothesis children reported substantially more group than personal discrimination, *t* (576) = 11.417, *p* < 0.001. Next, both personal and group discrimination showed small to moderate negative correlations with to self-esteem and national identification (Cohen 1988). The relation with self-esteem seemed to be stronger for personal discrimination and the relation with national identification seemed to be stronger for group discrimination. However, these differences were not substantial as the Group/Personal Discrepancy was unrelated to both of the outcome variables. Closeness with the teacher was positively related to national identification and self-esteem, and negatively to children’s overall perceptions of discrimination. Teachers’ implicit attitude toward Muslims versus non-Muslims was strongly and negatively related to national identification but not significantly associated with the other variables. Finally, there was a positive correlation between teacher religion and teachers’ implicit attitude indicating that Muslim teachers were more positively inclined toward Muslims (versus non-Muslims) compared to their non-Muslim colleagues. Further inspection showed that Muslims teachers had an implicit preference for Muslims over non-Muslims (*M* = 0.46, *SD* = 0.33; *t*(19) = 6.116, *p* < .001) whereas non-Muslim teachers did not prefer one of the groups (*M* = −0.11, *SD* = 0.63; *t*(14) = −.697, *p* = .497).

Table 1 also reports the intraclass correlations as an indication of the proportion of variance situated at the classroom level. For the variables assessed at the student level, the proportion of variance situated at the classroom level was relatively low. Hence, children in the same classroom were relatively dissimilar with respect to perceptions of discrimination, closeness with the teacher, national identification, and self-esteem.

### Multilevel Models

Next, three multilevel models were specified to test the hypotheses. In the first model only the covariates entered as predictors of national identification and self-esteem. Results are shown in Table 2. The fit indices indicate that this model fitted the data well and model fit improved further with each subsequent model. The results regarding the covariates indicate that girls reported higher levels of national identification and self-esteem than boys. Students’ ethnicity was also examined as a predictor of national identification and self-esteem. Moroccan students were considered as the reference group in the analyses because they formed the largest ethnic group in our sample. Turkish students reported lower levels of national identification compared to Moroccan ones. Grade was entered as a class-level covariate. Grade was not significantly associated with either national identification or self-esteem.

**Table 2:** Results of multilevel analyses for national identification and global self-esteem (unstandardized estimates)

	Model 1		Model 2		Model 3	
	Nat. Id.	Self-esteem	Nat. Id.	Self-esteem	Nat. Id.	Self-esteem
Covariates level 1						
Girl (ref = boy)	.32**	.15*	.26**	.10	.27**	.10
Ethnicity Turkish (ref = Moroccan)	−.29**	−.10	−.25**	−.15*	−.22*	−.14*
Ethnicity Other (ref = Moroccan)	−.18	.06	−.18	.07	−.15	.07
Covariates level 2						
Grade (year)	.00	.06**	−.02	.01	.03	.03
Predictors level 1						
Overall discrimination			−.11**	−.14***	−.12**	−.14***
Group/personal discrepancy			.01	.04	.02	.04
Closeness			15**	.19***	.14**	.19***
Overall discrimination × closeness			−.08	.02	-.08^*^	.02
Group/personal discrepancy × closeness			−.04	.00	-.04	.00
Predictors level 2						
Teacher religion (Muslim vs. non-Muslim)					−.16*	−.07
Teacher’s implicit religious attitude					−.05	−.03
Teacher religion × Teacher’s implicit religious attitude					.26*	−.10
Variance						
Level 1	.801	.359	.751	.310	.744	.311
Level 2	.005	.014	.007	.006	.002	.004
R^2^			5.96%	15.28%	7.44%	15.55%
Model fit						
AIC	4795.356		2454.917		2447.104	
SRMR Level 1	0.077		0.009		0.009	
SRMR Level 2	0.328		0.041		0.008	

^*^p < .05, **p < .01, ***p < .001

#### Discrimination and teacher closeness

In Model 2, perceived overall discrimination, group-personal discrepancy, closeness with the teacher, and the interaction between closeness and the former variables were added as predictors (see Table 2). Consistent with H2a and H3a, children’s overall perceptions of religious discrimination predicted lower self-esteem and lower national identification. The corresponding standardized coefficients indicated that the effect sizes were small to medium for the effect of discrimination on self-esteem (ES = −0.23) and small for the effect of discrimination on national identification (ES = −0.12). However, and contradicting H2b and H3b, the discrepancy between group and personal discrimination did not have any effect on either self-esteem or national identification. H4 and H5 stated that children who shared a close relationship with their primary teacher would have higher self-esteem and a stronger sense of national identification. These hypotheses were confirmed, and the corresponding effect sizes (ES = 0.14 and ES = 0.28) indicate small to moderate effects.

**Fig. 1:**
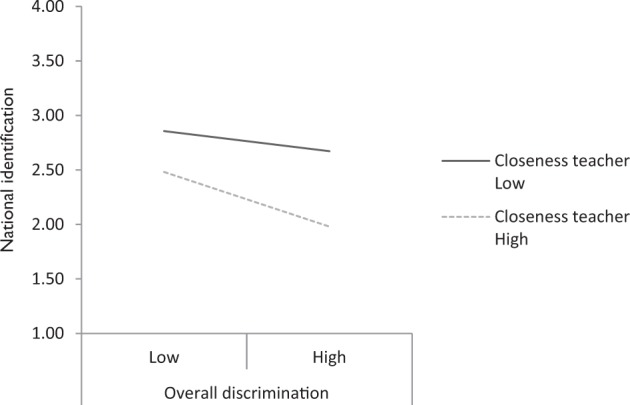
Regression lines of the interaction between perceived group discrimination and closeness with the teacher for the prediction of students’ national identification. *Note*: Low and high scores for perceived group discrimination and closeness are calculated based on scores 1 SD below or above the mean of these variables

Next, there was no support for the hypotheses that closeness diminished the negative effects of overall discrimination on self-esteem and national identification (H6a and H7a) or the effects of the discrepancy (H6b and H7b). In fact, there was a marginally significant interaction (*p* = 0.080) between closeness and the group/personal discrepancy in the prediction of national identification that was negative rather than positive. This interaction was further inspected as it became significant in Model 3. As shown in Fig. 1, a good relationship with the teacher appeared to increase rather than diminish the negative impact of perceived discrimination on students’ sense of national identification.

#### Teacher religion and implicit attitude

**Fig. 2:**
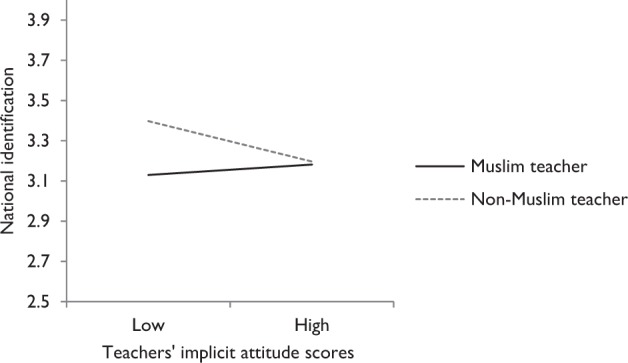
Regression lines for students of Muslim and non-Muslim teachers for the prediction of students’ national identification. *Note:* Low and high scores for teachers’ implicit attitudes for Muslims versus non-Muslims are calculated based on scores 1 SD below or above the mean of teachers’ IAT scores

In Model 3, teachers’ religion and implicit attitude toward Muslims versus non-Muslims were added, as well as the interaction between these two variables. It was expected that children would report a stronger sense of national identification if their teacher was a non-Muslim (H8). The results of Model 3 confirm this relationship for national identification (ES = −0.60). Hence, when their teacher was a non-Muslim, students reported a higher level of national identification. H9 and H10 stated that students would have higher self-esteem and a weaker sense of national identification, respectively, if their teachers had a more positive implicit attitude toward Muslims. These main effects were not significant. However, for national identification there appeared to be an interaction with teachers’ implicit attitude (ES = 0.56). This interaction is depicted in Fig. 2 and indicates that only the implicit attitude of the non-Muslim teachers had a negative effect. Note, that although the standardized estimates suggest strong effects of the teacher variables, they only pertain to the between level at which the outcome variables have limited variance. The total variance of national identification explained by adding teacher religion, implicit attitude, and the interaction is 1.48% (see also Table 2).

#### Additional analyses: Teachers’ ethnic background

Given the large overlap between teachers’ religion and ethnic background (90.0% of the Muslim teachers in our sample were from a non-Dutch background) it was not possible to include both teacher religion and teacher ethnicity in the same model. Yet, we did examine whether the effects for teachers’ ethnicity were similar to those of these religion. As expected, we found that non-Dutch teachers had a more positive attitude toward Muslims versus non-Muslims (*M* = 0.47, *SD* = 0.36) compared to Dutch teachers (*M* = −0.13, *SD* = 0.60), *t*(21) = −3.387, *p* < .003. Furthermore, we also ran Model 3 with teacher ethnicity instead of teacher religion as a predictor to examine the effects of teacher ethnicity on students’ national identification and self-esteem, and we obtained similar results. That is, we found a significant effect of teachers’ ethnicity on students’ national identification, with students of Dutch teachers reporting higher national identification (*b* = .16, *p* = 0.033) and we found a significant interaction (but no significant main effect of teachers’ implicit attitude) of teacher ethnicity and teachers’ implicit attitude on national identification (*b* = -0.24, *p* = 0.010), which indicated that particularly Dutch teachers’ implicit attitude toward Muslims versus non-Muslims is associated with students’ national identification. The main effects and interaction of teacher ethnicity and teachers’ implicit attitude on self-esteem were not significant (*p* > 0.05). Hence, as could be expected based on the substantial overlap in teachers’ religion and ethnic background, both teacher ethnicity and teacher religion yielded similar results.

## Discussion

There is considerable debate about the contribution and effects of Islamic education in Western societies. Islamic schools provide Muslim children with a safe context where they can openly practice their religion and do not have to worry about religious discrimination and Islamophobia. Yet children who attend these schools have considerably less opportunities to meet peers from other- or non-religious backgrounds, and theoretically this could undermine their future integration in societies where Muslims are a stigmatized minority (Thijs and Verkuyten 2014). Researchers can test the empirical foundations for these and other arguments by systematically comparing the effects of Islamic versus non-Islamic education. However, given the growing presence of Islamic schools in many Western societies (Dronkers 2016; Maussen and Bader 2014), it is more practical and perhaps more interesting to know how different factors *within* these schools affect how children feel about themselves and the country they live in. This study is the first quantitative research to provide this much needed knowledge, and educators could use it insights to help Islamic school students deal with religious discrimination and foster their self-esteem and national identification.

The current results show that Islamic school children report substantially more discrimination against their group than against themselves. This is in line with the Personal/Group Discrimination Discrepancy (Taylor et al. 1990) but also consistent with the notion that Islamic schools can partly shelter Muslim children from personal discrimination. Next, children’s overall perceptions of religious discrimination were associated with lower self-esteem, but unexpectedly the difference between group and personal discrimination (group/personal discrepancy) had no significant effect which indicates that the impact of the latter was not substantially stronger than that of the former. These findings indicate that religious discrimination undermines Muslim children’s self-evaluations presumably because it conveys a negative message about their religious identity which they come to internalize (Harter 1999). However, the results do not support the reasoning that perceiving more discrimination against one’s fellow in-group members versus the self promotes self-esteem as this helps people to feel good about themselves as individuals (see Bourguignon et al. 2006). Perhaps this reasoning is adequate but simply does not apply to Muslim children. Earlier research has reported very high rates of a religious identification among Muslim youth in the Netherlands (Verkuyten et al. 2012), and as a result it may be difficult for them to separate the implications of what happens to them personally from what happens to their co-religious peers. The relatively strong correlation between perceived group and perceived personal discrimination in the present study (*r* = 0.60) as compared to other studies among minority youth (*r* = 0.48, Stevens and Thijs in press; *r* = 0.41, Armenta and Hunt 2009) is consistent with this interpretation. However, future research should directly test this by including a religious identification measure.^4^

The analyses also supported the hypothesis that Muslim children’s perceptions of religious discrimination undermine their national identification. Unexpectedly, there was no effect of the group/personal discrepancy indicating that group discrimination is not more harmful than personal discrimination in this respect. Again, this might be due to children’s strong religious identification, and future research should test this interpretation. Still, consistent with the rejection-disidentification model (Jasinskaja-Lahti and Liebkind 2009), it is more difficult for Muslim children in a non-Muslim country to feel a connection to that country and its “original” inhabitants, if they have the awareness that they or their religious in-group peers are not accepted there (see also Fleischmann and Phalet 2018).

Next, it was found that Islamic school students who experienced a close, personal bond with their teachers had higher self-esteem and a stronger sense of national identification. Islamic school teachers are in a unique position to counteract the negative effects of discrimination for their students: As potential secondary attachment figures (Verschueren and Koomen 2012), they can help their students feel good about themselves (Ryan et al. 1994), and as representatives of the national educational system (Dronkers 2016) they can stimulate a sense of connection to society at large. Yet, unexpectedly, relational closeness did not diminish the negative impact of perceived discrimination. First, perceived discrimination was found to be associated with lower self-esteem regardless of children’s bond with their teachers. This results seems to contradict findings from an earlier study which showed that positive relationships with teachers protected immigrant students’ self-esteem against the negative impact of perceived ethnic peer harassment (Özdemir and Stattin 2014). Importantly, however, that previous study was conducted in public schools. In principle, public schools are open to different groups of students, which implies that part of students’ discrimination experiences can take place within the school environment itself. The current research took place within Islamic schools where religious discrimination of Muslim children is extremely unlikely. To the extent that Islamic school students perceive instances of religious discrimination, this most likely happens outside their school context. Apparently, teachers are less relevant for helping children deal with these negative out-of-school experiences.

Second, children’s closeness with their teachers appeared to strengthen rather than diminish the negative impact of their overall perceptions of discrimination on their national identification. A possible explanation for this unexpected finding is that this relationship provides Islamic school children with a sense of safety and local belonging which allows them to turn away from a society they perceive as hostile. Thus, these children could “afford” national disidentification in response to perceived rejection. Still, this should not be taken to mean that close relationships with Islamic school teachers move children away from mainstream society. Relational closeness was negatively related to children’s perceptions of discrimination, and its overall effect on national identification was positive.

Next to children’s subjective perceptions of the student-teacher relationship, the effects of teachers’ religious background and implicit religious attitude were examined. In Islamic schools, children have no opportunities to interact with non-Muslim peers, whereas these interactions could contribute to a shared national identity (Knifsend et al. 2017). However, it was found that children with a non-Muslim teacher reported more national belonging than students with a Muslim teacher, which indicates that non-Muslim teachers have a considerable role to play as a bridge to society for Islamic school students in a non-Muslim country. Apparently, contact with these teachers makes it easier for them to feel part of a common national in-group that is largely composed of religious out-group members (see Dovidio et al. 2007).

Unexpectedly there was no main effect of teachers’ implicit religious attitude on children’s national identification. However, there was a significant interaction with teachers’ religious background, and the religious attitude of non-Muslim teachers was associated with a weaker sense of national identification in their students. Presumably, teachers with a relatively positive attitude toward Muslims communicated a comparatively less positive evaluation of non-Muslim natives to their students, and this evaluation had a negative impact on children’s identification with their non-Muslim country. The fact that the implicit attitude of only the non-Muslim teachers was found to matter could mean that Islamic school children are more attentive to the religious evaluations of the non-Muslim majority and consider them to be more knowledgeable about that group. It also means that the positive effect of having a non-Muslim teacher can be undone if he or she has a comparatively less positive attitude toward non-Muslim natives. Possibly, non-Muslim teachers with such an attitude are seen as less exemplary for “the non-Muslim out-group”, which undermines the contact potential of children’s interactions with these teachers, and hence their contribution to the development of a common national identity (see Thijs and Verkuyten 2012). Not surprisingly, Muslim teachers scored higher on the implicit attitude measure than their non-Muslim colleagues, but their religious preference did certainly not undermine the national identification of their students.

Finally, the analyses did not support the expectation that the children had higher self-esteem if they a had a teacher with a comparatively positive attitude toward Muslims versus non-Muslims. Perhaps, these teachers only demonstrated a less positive attitude toward non-Muslim natives a suggested above. And this was probably irrelevant for how the children evaluated themselves. Another possibility is that the school environment of the children explicitly supported their religious identity which would make their self-evaluations less dependent on the religious attitude of their teachers. Future research is necessary to test such interpretations.

In evaluating the present study, some qualifications and limitations need to be considered. First, as indicated above, the (non)impact of teachers’ implicit attitude can be interpreted in different ways. Like many other IAT measures (Greenwald et al. 2009), the present instrument juxtaposed teachers’ evaluations of two groups (Muslims versus non-Muslims). This means that one cannot establish whether the effects of teachers’ implicit attitude are due to their positive evaluations of Muslims or rather their less positive evaluations of non-Muslims. Future studies should use separate measures to disentangle these different implicit attitudes. Second, the use of cross-sectional data undermines the ability to make causality claims. For example, one cannot rule out the possibility that Muslim children with lower self-esteem or a weaker sense of national identification are more likely to perceive discrimination directed against themselves or their group, respectively. It makes theoretical sense to regard self-esteem and national identification as outcomes of perceived discrimination, and it is very unlikely that teachers’ own religious background and attitude depend on their students’ national identification and self-esteem. However, future research should use cross-lagged models to test the direction of effects assumed in the present study. Third, this study relied on children’s perceptions of the quality of the student-teacher relationship. This choice can be defended by stressing the psychological importance of children’s relationship experiences (Koomen and Jellesma 2015), but it would be worthwhile to replicate the present findings with teachers’ reports of the student-teacher relationship. Finally, this study took place in the Netherlands where primary school children tend to have the same single teacher across the whole year. It is important to replicate its findings among Islamic school children in other Western countries.

Despite its limitations, the present study has some practical implications. For one, Islamic schools should actively promote religious diversity amongst their teaching staff in order to stimulate a sense of national identification in their students. And importantly, the non-Muslim teaching staff should not be negatively inclined toward non-Muslims natives to have this positive impact. As the present findings indicate, non-Muslim teachers who chose to work in Islamic schools are unlikely to prefer non-Muslims over Muslims. Yet it could be worthwhile for these schools to overcome this “self-selection”, and also employ non-Muslim teachers who are slightly biased toward “their own group”, just as their Muslim colleagues. Next the current findings indicate that Islamic school teachers could be trained in helping their students cope with religious prejudice. Close relationships with their teachers allow these children to feel good about themselves in spite of the discrimination against their group. Yet teachers should be made aware of the possibility that these relationships could inadvertently facilitate a process of national disidentification for students who perceive much of this discrimination. Thus the challenge is to provide a safe base that allows children toward rather than away from mainstream society.

## Conclusion

The present study makes a unique contribution to the literature by examining the self-esteem and national identification of Islamic school students in a highly secular country, and by studying the impact of perceived religious discrimination and the roles of the student-teacher relationship as well as teachers’ religious background and implicit attitude. The results clearly show that perceived religious discrimination threatens children’s self-esteem, just as ethnic discrimination does (see Thijs and Verkuyten 2017), and undermines their identification with a country where many people hold negative attitudes toward their religion (see Fleischmann and Phalet 2018). Importantly, it does not seem to matter whether this discrimination is directed against children themselves or against their in-group peers. Thus in the case of Muslim children there are no relative “benefits” of perceived group discrimination as have been found in studies on ethnic and gender discrimination (Bourguignon et al. 2006; Stevens and Thijs 2018). The present findings also paint a nuanced picture of the way Islamic school teachers affect their students’ self-esteem and national identification. Children who shared a close bond with their teachers reported higher self-esteem, and thus, from a resilience perspective (Motti-Stefanidi and Masten 2013), the student-teacher relationship can be regarded as a promotive factor that counteracts the negative impact of discrimination. Yet at the same time, a strong and positive student-teacher relationship appears to facilitate a process of rejection-disidentification whereby perceived religious discrimination thwarts children’s sense of national identification. Additionally, the presence of non-Muslim teachers facilitates children’s identification with their non-Muslim country, but this effect can be undone if these teachers have a preference for Muslims over non-Muslim natives. It may be rather difficult for Islamic schools in non-Muslim countries to prepare their students to function in mainstream society, yet the insights from the current study may be useful.

## Footnotes

1 Note that his difference involves a *group/personal discrepancy* rather than the Personal/Group Discrepancy (Taylor et al. 1990).

2 The IAT by Van den Bergh et al (2010) measured teachers’ implicit attitudes toward ethnic minority students versus ethnic minority students and required participants to classify the names as “Dutch” or “Turkish/Moroccan”. As the Muslim population in the Netherlands almost exclusively consists of people with an ethnic minority background, mainly originating from Morocco and Turkey (Central Bureau of Statistics 2016), the names of ethnic minority students versus Dutch students could also be categorized with the “Muslim” and “non-Muslim” labels.

3 Years of teaching experience was not significantly related to the outcome measures, and therefore not included as a control variable.

4 The present dataset included such a measure. However, it could not be used due to a severe restriction of range (mean item scores >4.9 on a scale from 1–5) which indicates that the religious identification of the children was very strong indeed.
